# Aerobic exercise reduces oxidative damage and improves heart function in diabetic rats with heart failure: No extra effect from photobiomodulation

**DOI:** 10.1007/s40200-026-01924-5

**Published:** 2026-04-13

**Authors:** Naira Helena Bohrer Scherer, Alan Christhian Bahr, Lucas Capalonga, Gilson Pires Dorneles, Pedro Dal Lago

**Affiliations:** 1 Laboratory of Experimental Physiology, Universidade Federal de Ciências da Saúde de Porto Alegre (UFCSPA), Porto Alegre, RS Brazil; 2 Graduate Program in Rehabilitation Sciences, Universidade Federal de Ciências da Saúde de Porto Alegre (UFCSPA), Porto Alegre, RS Brazil; 3 Universidade do Vale do Taquari, Lajeado, RS Brazil; 4 Laboratory of Imunology, Universidade Federal de Ciências da Saúde de Porto Alegre (UFCSPA), Porto Alegre, RS Brazil; 5 Graduate Program in Rehabilitation Sciences (PPG-CR), Universidade Federal de Ciências da Saúde de Porto Alegre (UFCSPA), Rua Sarmento Leite, 245, Porto Alegre, RS 90050-170 Brazil

**Keywords:** Aerobic exercise, Photobiomodulation, Heart failure, Type 2 diabetes mellitus

## Abstract

**Purpose:**

The coexistence of diabetes mellitus (DM) and heart failure (HF) worsens clinical outcomes and complicates therapeutic strategies. This study investigated the effects of aerobic exercise (AE), with or without photobiomodulation (PBM), on cardiac function, glycaemic control, inflammation, and oxidative stress in diabetic rats with HF.

**Methods:**

Fifty rats were initially enrolled. Ten animals died during myocardial infarction surgery (20% mortality), and among the 40 survivors, exclusions based on small infarct size resulted in a final sample of 30 rats for analysis (HFDM: *n* = 11; HFDM + AE: *n* = 12; HFDM + AE+PBM: *n* = 7). AE was performed on a treadmill for eight weeks. PBM (850 nm, 4.4 J) was applied bilaterally to the gastrocnemius muscle after each AE session.

**Results:**

AE was associated with increased exercise duration, running distance, and maximal speed compared with HFDM (*p* < 0.0001). AE also improved haemodynamic indices (+ dP/dtmax and -dP/dtmin; *p* < 0.001), reduced left ventricular posterior wall thickness (*p* = 0.0001), lowered fasting glycaemia (*p* = 0.032), and enhanced antioxidant defenses, including increased catalase and glutathione peroxidase activity and reduced plasma lipid peroxidation (TBARS; *p* < 0.0001). The addition of PBM did not confer further benefits beyond those induced by AE (*p* > 0.05).

**Conclusion:**

AE improved functional capacity, cardiac structural and haemodynamic parameters, fasting glycaemia, and oxidative stress in rats with HF and DM. Under the conditions applied, PBM did not provide additional benefits, indicating that further studies are needed to optimize PBM protocols and clarify its potential role as an adjunct therapy in HFDM.

## Introduction

Heart failure (HF) and type 2 diabetes mellitus (DM) are chronic diseases with growing global prevalence, that frequently coexist, compounding their individual clinical impact [1–3]. DM is a major risk factor for the development of HF, contributing to a worse prognosis and higher mortality rates [2]. The coexistence of these conditions leads to profound systemic alterations, including chronic hyperglycaemia, mitochondrial dysfunction, increased oxidative stress, and impaired calcium homeostasis. Collectively, these disturbances promote structural and functional cardiac damage, systemic inflammation, endothelial dysfunction, muscle atrophy, and exercise intolerance [4–6].

Exercise intolerance is the hallmark symptom of HF associated with DM (HFDM), and results from multiple pathophysiological mechanisms, including reduced cardiac output, impaired skeletal muscle perfusion, autonomic imbalance, and increased neurohumoral activation, particularly involving catecholamines and angiotensin II [7]. Together, these maladaptations contribute to dyspnoea, fatigue, reduced quality of life, and increased risk of adverse clinical outcomes. Experimental models of HF are widely used in the literature and typically define systolic HF based on well-established criteria, which involve a reduction in left ventricular (LV) ejection fraction (EF) to ≤ 50% and presence of extensive ischaemic injury (≥ 30%) [8]. These features ensure that the resulting structural and functional changes closely resemble those observed in humans, providing a robust framework for the investigating potential therapeutic interventions.

Non-pharmacological interventions have increasingly gained attention as complementary therapeutic strategies in HFDM. Among these, aerobic exercise (AE) is one of the most effective approaches, with well-documented benefits for improving functional capacity, vascular and muscular adaptations, autonomic regulation, and myocardial performance [9, 10]. In addition, AE enhances antioxidant defenses by reducing the production of reactive oxygen species (ROS) and upregulating key antioxidant enzymes, including catalase (CAT), glutathione peroxidase (GPX1), and superoxide dismutase (SOD). Concurrently, AE exerts anti-inflammatory effects through the modulation of cytokine signaling pathways [11–13].

Photobiomodulation (PBM) has recently emerged as a promising complementary therapeutic approach. PBM is a non-invasive, non-thermal light-based therapy that uses specific wavelengths of red and near-infrared light to trigger photochemical reactions in biological tissues through mitochondrial chromophores, particularly cytochrome c oxidase [14]. Evidence from diabetic and cardiovascular experimental models indicates that PBM reduces oxidative stress, as reflected by lower levels of thiobarbituric acid reactive substances (TBARS) [15], and modulates inflammatory responses by decreasing tumor necrosis factor alpha (TNF-α) and interleukin-6 (IL-6), while increasing interleukin-10 (IL-10) [16]. Furthermore, PBM has been associated with improvements in muscle performance and endurance in experimental models, particularly when applied in combination with exercise training [17–19]. The gastrocnemius muscle has been identified as a relevant therapeutic target in HF, particularly due to its involvement in skeletal muscle atrophy and dysfunction that contribute to reduced exercise capacity [18]. Accordingly, PBM applied specifically to the gastrocnemius muscle may elicit systemic effects, with potential downstream repercussions on cardiac function. Despite these advances, the potential additive or synergistic effects of AE and PBM in HFDM remain poorly understood. Therefore, this study aimed to investigate the effects of AE alone and in combination with PBM on haemodynamic function, functional capacity, glycaemic control, inflammatory profile and oxidative stress in a rat model of HFDM induced by myocardial infarction (MI).

## Methods

### Ethical approval

All procedures were conducted in accordance with the Animal Research Reporting of In Vivo Experiments (ARRIVE) guidelines and complied with the Guide for the Care and Use of Laboratory Animals [20]. The experimental protocol was approved by the Ethics Committee on Animal Use of the Universidade Federal de Ciências da Saúde de Porto Alegre (UFCSPA) under protocol number 655/19.

### Animals

Fifty male Wistar rats (approximately 30 days old) were obtained from the UFCSPA Animal Breeding Unit. Animals were housed three per cage with ad libitum access to food and water, under controlled temperature conditions (22 °C) and a 12 h light–dark cycle. Importantly, MI was performed when the animals were approximately 3 months old, an age widely considered representative of adulthood in rats.

### Experimental design

**Fig. 1:**
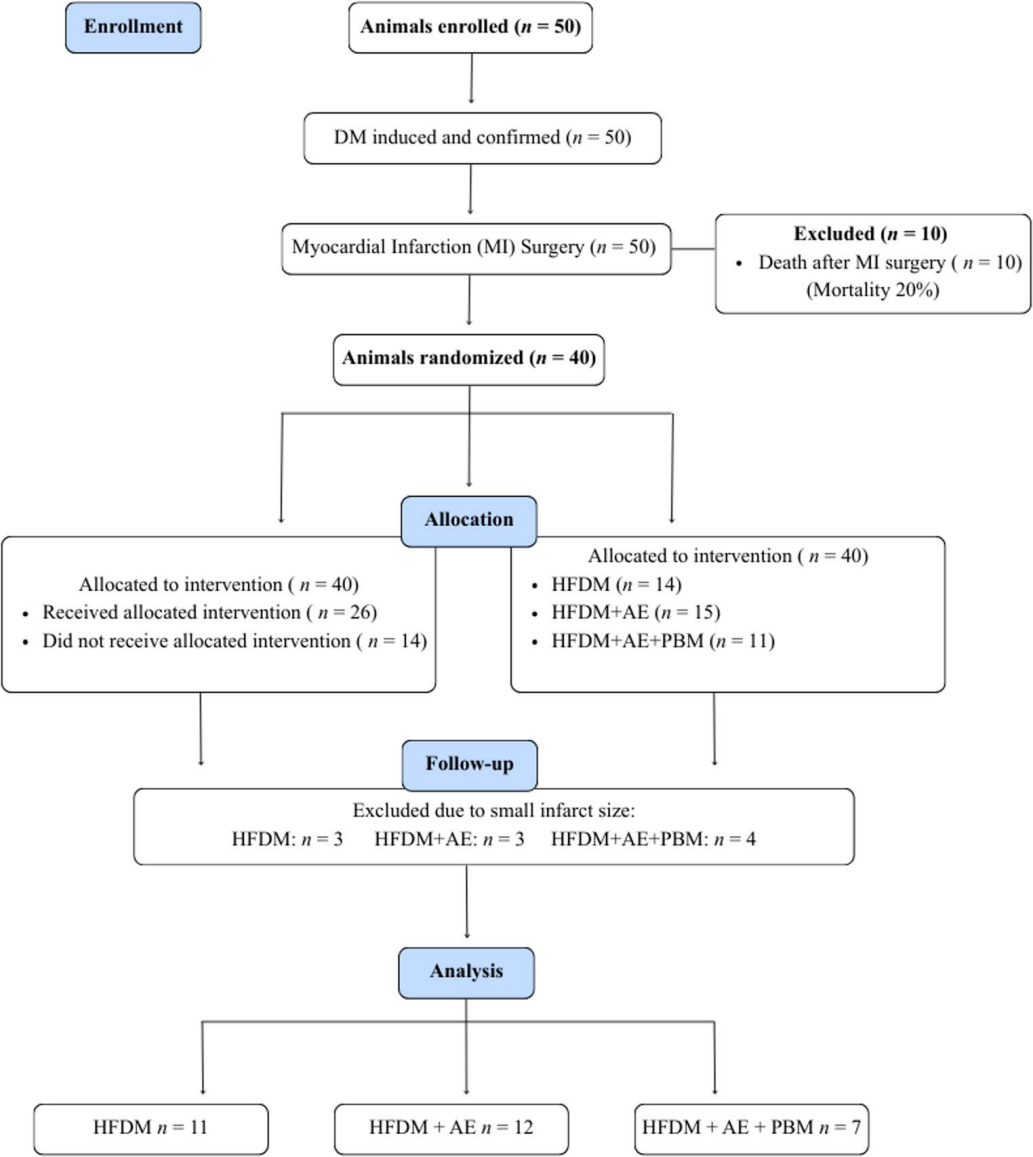
Experimental flowchart of the study design. The diagram illustrates animal enrollment, induction of type 2 diabetes mellitus, myocardial infarction (MI) surgery, randomization, group allocation, follow-up, and final analysis. Fifty animals underwent diabetes induction and MI surgery; ten animals died after MI surgery (20% mortality). Forty animals were randomized and allocated to the experimental groups. During follow-up, animals were excluded due to small infarct size (HFDM: *n* = 3; HFDM + AE: *n* = 3; HFDM + AE+PBM: *n* = 4). Final sample sizes used for analysis were HFDM (*n* = 11), heart failure associated with type 2 diabetes mellitus; HFDM + AE (*n* = 12), heart failure associated with type 2 diabetes mellitus treated with aerobic exercise; and HFDM + AE+PBM (*n* = 7), heart failure associated with type 2 diabetes mellitus treated with aerobic exercise combined with photobiomodulation. Abbreviations: MI, myocardial infarction

**Fig. 2:**
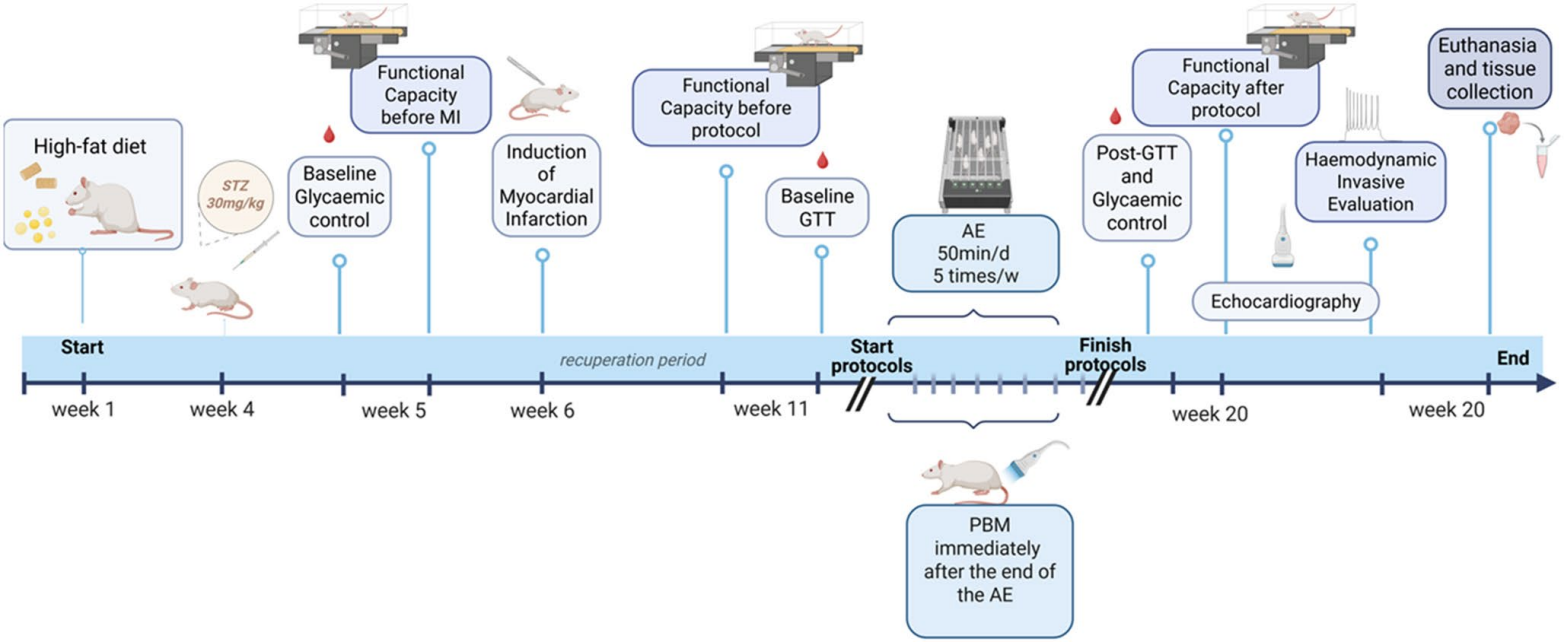
Experimental timeline of the study protocol. The timeline illustrates the sequence of experimental procedures, including induction of type 2 diabetes mellitus by streptozotocin (STZ), confirmation of glycaemic status, myocardial infarction (MI) surgery, assessment of functional capacity before MI and before the intervention, baseline glucose tolerance testing (GTT), initiation and completion of the aerobic exercise (AE) and photobiomodulation (PBM) protocols, post-intervention GTT and glycaemic assessment, echocardiographic evaluation, invasive haemodynamic assessment, and tissue collection at the end of the study. The duration of each experimental phase and the timing of all evaluations are indicated along the timeline

All rats underwent induction of type 2 DM followed by MI. Of the 50 rats initially enrolled, 10 died during MI surgery (20%), leaving 40 animals eligible for randomization. After a 4-week recovery period and confirmation of eligibility, animals were randomized using a simple randomization procedure without block stratification. Randomization was performed by an investigator not involved in data collection, resulting in unequal group sizes: HFDM (HF + DM, no intervention), HFDM + AE (HF + DM + aerobic exercise), and HFDM + AE+PBM (HF + DM + aerobic exercise + photobiomodulation). Based on the predefined criterion of MI size > 30%, 3 rats in HFDM group, 3 in HFDM + AE group, and 4 in HFDM + AE+PBM group were excluded. This result in a final sample of 30 animals (HFDM: *n* = 11; HFDM + AE: *n* = 12; HFDM + AE+PBM: *n* = 7) (Fig. 1). The study was designed to investigate the incremental effects of PBM when combined with AE, rather than to evaluate PBM as an isolated intervention. Functional capacity was assessed before MI and both before and after the intervention period. Echocardiography was performed after 8 weeks of intervention, with left ventricular (LV) ejection fraction (EF) as the primary outcome for functional characterization of MI severity. However, EF was not used as a pre-specified inclusion or exclusion criterion prior to initiation of the experimental interventions. Animal inclusion was based solely on histological infarct size criteria. Glycaemic assessments, including fasting blood glucose and glucose tolerance test (GTT), were conducted before MI and both before and after the interventions. Body mass was monitored weekly throughout the study. At the end of the experimental period, haemodynamic evaluation, euthanasia, and tissue collection were performed (Fig. 2). Investigators responsible for functional testing, echocardiographic assessments, haemodynamic measurements, and biochemical analyses were blinded to group allocation throughout data acquisition and analysis.

### Induction of type 2 diabetes mellitus

The protocol described by Zhang et al. [21] was used to induce type 2 DM. Rats were fed a high-fat diet (58.3% fat, 24.5% carbohydrate, and 17.2% protein) supplemented with albumin, amino acids, and mineral powder. The diet was prepared weekly and stored at 4–8 °C. After 4 weeks of dietary intervention, animals received intraperitoneal (i.p.) injection of streptozotocin (STZ, 30 mg/kg; Sigma-Aldrich, St. Louis, MO, USA). A fasting glucose threshold > 140 mg/dL was adopted, in accordance with the characteristics of the high-fat diet plus low-dose STZ model and previous validation studies using similar rat strains and glucometer-based whole-blood measurements. Rats presenting fasting glucose levels < 140 mg/dL one week after the first STZ injection received a second STZ dose (30 mg/kg, i.p.; Sigma-Aldrich, St. Louis, MO, USA). This protocol induces partial β-cell dysfunction combined with diet-induced insulin resistance, closely resembling type 2 DM [21].

#### Glycaemic control

Fasting blood glucose levels were measured at week 5 (post-diet) and at week 20 (end of intervention period) using tail vein blood and a portable glucometer with test strips (Accu-Chek^®^ Performa, Roche Diagnostics, Basel, Switzerland).

#### Glucose tolerance test (GTT)

After 12 h fasting period, rats received intraperitoneal injection of a 40% glucose solution (2 g/kg). Tail vein blood samples were collected at 0, 30, 60, 90, and 120 min. and glycaemic response was expressed as area under the curve (AUC). The GTT was performed one week before the start of the experimental protocols and one day after the final session of AE and PBM, all analysis were performed using tail blood and test strips (Accu-Chek^®^ Performa; Roche Diagnostics, Basel, Switzerland).

### Induction of myocardial infarction

Six weeks after the initiation of the high-fat diet, animals underwent MI induction by permanent ligation of the left coronary artery. The procedure was performed under anaesthesia with 2% isoflurane, animals were intubated, and mechanically ventilated (SamWay VR 15, Montevideo, Uruguay), at a respiratory rate of 60 breaths min^− 1^ with an inspired oxygen fraction of 100%, as previously validated in our laboratory [22, 23]. Following thoracotomy, the left coronary artery was ligated using a 6 − 0 mononylon suture. The chest was then closed, and pneumothorax drained. During the first 48 h post-surgery, animals received subcutaneous ketoprofen (0.15 mg/kg) for postoperative analgesia and a single intraperitoneal dose of penicillin (20,000 U). Animals were allowed to recover for 4 weeks before further experimental procedures. All the surgeries were performed by the same investigator.

### Assessments

#### Functional capacity

Four weeks after MI surgery, animals were acclimated to the treadmill exercise and testing environment for one week. Each acclimation session lasted 5 min/day, at a speed 10 m/min and was performed three times per week [19]. During the subsequent week, rats were subjected to an incremental treadmill exercise protocol until exhaustion. The incremental treadmill test, which began at 5 m/min with speed increments of 5 m/min every 3 min until exhaustion, was performed at three time points: pre-MI, pre-intervention, and post-intervention. Testing was conducted in a small animal treadmill, connected to an analyser apparatus (AVS Projects, São Carlos, SP, Brazil) allowing continuous monitoring of maximal speed, total distance covered, and running time until exhaustion [17, 19]. Data from the analyser and treadmill were imported into dedicated software (AQCAD version 2.3.9.0; AVS Projects, São Carlos, SP, Brazil) for analysis.

#### Echocardiography

Twenty-four hours after the final session of the experimental protocol, the animals underwent non-invasive cardiac function assessment using a commercially available echocardiography (GE Vivid I; GE Medical Systems, Tel Aviv, Israel) equipped with an 8–13 MHz electronic transducer. All evaluations were performed by the same researcher, using standardized image planes and parameters validated for small-animal echocardiography. Although based on clinical echocardiography principles [24], methodological adaptations were applied according to protocols previously described for rodents [25]. Under inhalation anaesthesia (2% isoflurane), left ventricular volumes were estimated using a cubic model, and EF, fractional shortening (FS), relative wall thickness (RWT), and LV dimensions normalized to body mass were calculated. Only rats with EF < 50% were included for descriptive characterization of HF severity.

#### Invasive haemodynamic

Twenty-four hours after echocardiography, invasive haemodynamic assessment was performed. Anaesthesia was induced with ketamine (90 mg/kg, i.p.) and xylazine (12 mg/kg, i.p.), and a small incision was made in the anterior cervical region to allow insertion of a polyethylene catheter (PE-50) into the right carotid artery. Arterial pressure was initially recorded over a 5-min period by connecting the arterial cannula to a pressure transducer (Miniature Pulse Transducer RP-155, Narco Biosystems, Houston, TX, USA) coupled to a pressure amplifier (General Purpose Amplifier 4, Model 2, Stemtech, Hudson, WI, USA). The catheter was then advanced into the left ventricle, and ventricular pressure waveforms were recorded for an additional 5 min. Pressure signals were digitized using a data acquisition system (AT/Codas, Dataq Instruments, Akron, OH, USA) at a sampling rate of 2000 Hz. These data were used to calculate the maximum (+ dP/dtmax) and minimum (− dP/dtmin) rates of left ventricular pressure development, as well as left ventricular end-diastolic pressure (LVEDP) [23].

Prior to each experiment, the pressure transducer and acquisition system were calibrated according to the manufacturer’s instructions. All haemodynamic recordings were obtained using standardized protocols. To minimize operator-related variability, catheter insertion, signal acquisition, and data analysis were performed by a single experienced investigator blinded to group allocation. Haemodynamic parameters were averaged over multiple consecutive cardiac cycles to improve measurement reliability and reproducibility.

#### Morphometry and congestion

The right ventricle (RV) and LV were dissected and weighed separately. Organ mass-to-body mass ratios were calculated. The lungs and liver of each animal were weighed and then dehydrated at 80 °C for 48 h. After this period, the tissues were reweighed to determine wet-to-dry weight ratios as indices of tissue congestion [11].

#### Infarct size

Infarct size was determined exclusively at the end of the experimental protocol by histological planimetry. The infarcted area was quantified in scanned left ventricular sections using ImageJ software (version 1.53; National Institutes of Health, Bethesda, MD, USA) [26]. All infarct size measurements were performed by a researcher blinded to the haemodynamic data. Animals presenting small infarcts based on histological analysis were excluded according to predefined criteria.

#### Tissue collection

Immediately after the invasive haemodynamic assessment, the animals, still under anaesthesia, were euthanized by decapitation. The entire heart was collected, and the left and right ventricles, lungs, liver, gastrocnemius muscle, and plasma were separated. All samples were immediately frozen in liquid nitrogen and stored at − 80 °C for subsequent analysis.

#### Cytokine levels

Left ventricular tissue, plasma and gastrocnemius muscle were used to determine the concentrations of TNF-α and IL-10. Interleukin-1 Beta (IL-1β) was assessed in LV and gastrocnemius samples. Plasma was obtained by centrifugation at 664 x g for 5 min. LV and gastrocnemius muscle samples were homogenized in potassium phosphate buffer (KPi, pH 7.4) containing 4.08 g/L KH_2_PO_4_, 8.9 g/L KCl, 8.71 µg/mL phenylmethylsulfonyl fluoride (PSMF), 0.1 µg/mL aprotinin, 0.1 µg/mL leupeptin and 0.1 µg/mL pepstatin as proteases inhibitors, using a hand-held homogenizer. The homogenates were centrifuged at 12,000 × g (Mikro 220 R, Hettich Zentrifugen, Tuttlingen, Germany) for 60 min at 4 °C. The supernatants were collected, and TNF-α, IL-1β and IL-10 levels were determined by multiplex bead-based immunoassay using Milliplex™ MAP Rat Cytokine kits (Merck Millipore/Millipore Sigma, Burlington, MA, USA). All samples were run in duplicate, and mean values were expressed as pg/mg of protein for tissue samples and pg/mL for plasma [27].

#### Oxidative stress analyses

To assess oxidative stress, TBARS, GPX1 and CAT activities were measured in LV tissue, plasma and gastrocnemius muscle. These markers were selected to provide complementary information on redox status, with TBARS reflecting lipid peroxidation to cell membranes, while GPX1 and CAT activities indicate endogenous enzymatic antioxidant defense capacity. All analysis were performed using a spectrophotometer. TBARS levels were determined based on malondialdehyde (MDA) formation and expressed as nanomoles of MDA per milligram of protein (nmol MDA/mg protein). GPX1 and CAT activities were expressed as units of enzyme per milligram of tissue (U GPX1/mg tissue and U CAT/mg tissue, respectively). CAT activity was determined by monitoring H_2_O_2_ decomposition at 240 nm [28]. Lipid peroxidation for the TBARS assay was measured at 535 nm [29]. GPX1 activity was assessed by measuring nicotinamide adenine dinucleotide phosphate (reduced form; NADPH) consumption at 340 nm using tert-butyl hydroperoxide as the substrate [30].

### Interventions

#### Aerobic exercise

From week 6 post-MI, rats performed treadmill running for 50 min/day, at an initial speed of 10 m/min, 5 days/week, for 8 weeks. Training load was progressively increased every two weeks and prescribed at 50–70% of maximal running speed. This protocol was based on previous studies [9, 23] demonstrating that moderate-intensity aerobic training promotes cardiovascular and metabolic adaptations in rodent models of HF while maintaining safety and feasibility. One week before the start of the AE protocol, the animals underwent an adaptation period consisting of treadmill running for 15 min/day at 10 m/min, three times per week. Sedentary animals were handled in parallel with trained animals and placed on the treadmill for an equivalent period, without running to control for handling and environmental stress. Throughout the intervention, average running speed ranged from 10 to 15 m/min, corresponding to 50–70% of maximal running speed as reassessed biweekly. All animals completed the training protocol, and no exercise-related dropouts were observed.

#### Photobiomodulation

Immediately after each AE session, animals received bilateral (medial and lateral) PBM using a gallium-aluminum-arsenide (GaAlAs) laser (850 nm, 4.4 J, 29.2 s/limb; ISO 13485, model 2779, Chattanooga Group Intelect^®^). Irradiation was applied to the gastrocnemius muscles, based on prior evidence of systemic effects [31, 32], at medial and lateral sites of the muscle belly, approximately 3 cm distal to the onset of the paw, with one application point per muscle belly. All experimental groups underwent daily LLLT for 10 consecutive days, with the probe positioned in direct contact with the skin at a 90° angle and gentle pressure applied. Prior to irradiation, the skin was shaved and cleaned to reduce light reflection and refraction, thereby enhancing laser efficacy [16].

#### Sample size and statistical analysis

The initial sample size was calculated a priori based on the primary outcome (functional capacity, assessed by running speed) using G*Power software (version 3.1.9.2). Assuming a one-way ANOVA design, the analysis indicated that six animals per group would be sufficient to detect a large effect size (Cohen’s f = 1.66), corresponding to a minimum expected between-group difference of 4 m/min, with an α level of 0.05 and 80% statistical power. Data normality was assessed by Shapiro–Wilk test. Exercise related variables were analysed using generalized linear models with a gamma distribution and Bonferroni correction. Glycaemic and body mass data were analysed by two-way ANOVA followed by Tukey’s post hoc test. One-way ANOVA with Tukey’s post hoc test was applied to oxidative stress, inflammatory, haemodynamic, and morphometric variables. Statistical analyses were performed using SigmaPlot 12.0, GraphPad Prism 8.0, and SPSS version 22. Results are presented as mean ± standard deviation (SD), and graphical data are shown with 95% confidence intervals when indicated. Statistical significance was set at *p* < 0.05.

## Results

### Body mass and infarct characteristics

Overall mortality was 20%. Table 1 summarizes body mass, indices of cardiac hypertrophy, infarct size, pulmonary and hepatic congestion, and gastrocnemius muscle weight. At baseline, body mass and fasting blood glucose levels were comparable among groups (Table 1), and all animals exhibited fasting blood glucose levels > 140 mg/dL, confirming successful induction of diabetes. After the intervention period, final body weight was significantly lower in the HFDM + AE and HFDM + AE+PBM groups compared with the HFDM (no intervention) group (*p* < 0.05). Infarct size was assessed histologically at the end of the experimental protocol, and infarct size distribution did not differ significantly among groups, indicating comparable myocardial injury severity across experimental conditions (Table 1). Left ventricular EF was assessed after MI for functional characterization but was not used as a baseline inclusion variable prior to the interventions.

**Table 1:** Body mass and infarcted characteristics of type 2 diabetic and heart failure

Measurements	HFDM (*n* = 11)	HFDM + AE (*n* = 12)	HFDM + AE+PBM (*n* = 7)	*P*-value
Infarcted Area (%)	31.2 ± 6.5	33.6 ± 6.7	33.9 ± 10.2	0.695
Initial Body Mass (g)	94 ± 18.2	103.6 ± 26.9	115.7 ± 27.7	0.064
Final Body Mass (g)	498.3 ± 70	432.5 ± 55*	432.1 ± 56.1#	0.006
Blood Glucose Before (mg/dL)	179.3 ± 107.2	248.6 ± 117.6	237.8 ± 128.3	0.459
Blood Glucose After (mg/dL)	163.1 ± 70	163.3 ± 69.9	186.9 ± 112	0.812
Pulmonary Congestion (%)	77.9 ± 3.7	73.6 ± 22.4	69.8 ± 13.4	0.630
Hepatic Congestion (%)	68.1 ± 2.4	58.9 ± 9	68.8 ± 2.7	0.403
LV Weight (g)	1.2 ± 0.1	1.1 ± 0.2	0.9 ± 0.1	0.144
LV Hypertrophy (g)	2.4 ± 0.1	2.5 ± 0.3	2.4 ± 0.2	0.705
Gastrocnemius (g)	2.1 ± 0.2	1.7 ± 0.1	1.8 ± 0.3	0.086
Gastrocnemius/BW (mg/g)	3.6 ± 1.4	4.1 ± 0.5	4.3 ± 0.6	0.2585

Values are presented as mean ± SD. Statistical comparisons were performed using one-way ANOVA followed by Tukey’s post hoc test. Abbreviations: HFDM, heart failure associated with type 2 diabetes mellitus; HFDM + AE, heart failure associated with type 2 diabetes mellitus treated with aerobic exercise; HFDM + AE+PBM, heart failure associated with type 2 diabetes mellitus treated with aerobic exercise and photobiomodulation; LV, left ventricle; BW, body weight; gastrocnemius/BW, gastrocnemius muscle weight normalized to body weight. *p < 0.0001 vs. HFDM; #*p* = 0.009 vs. HFDM

### Glucose tolerance and glycaemic control

**Fig. 3:**
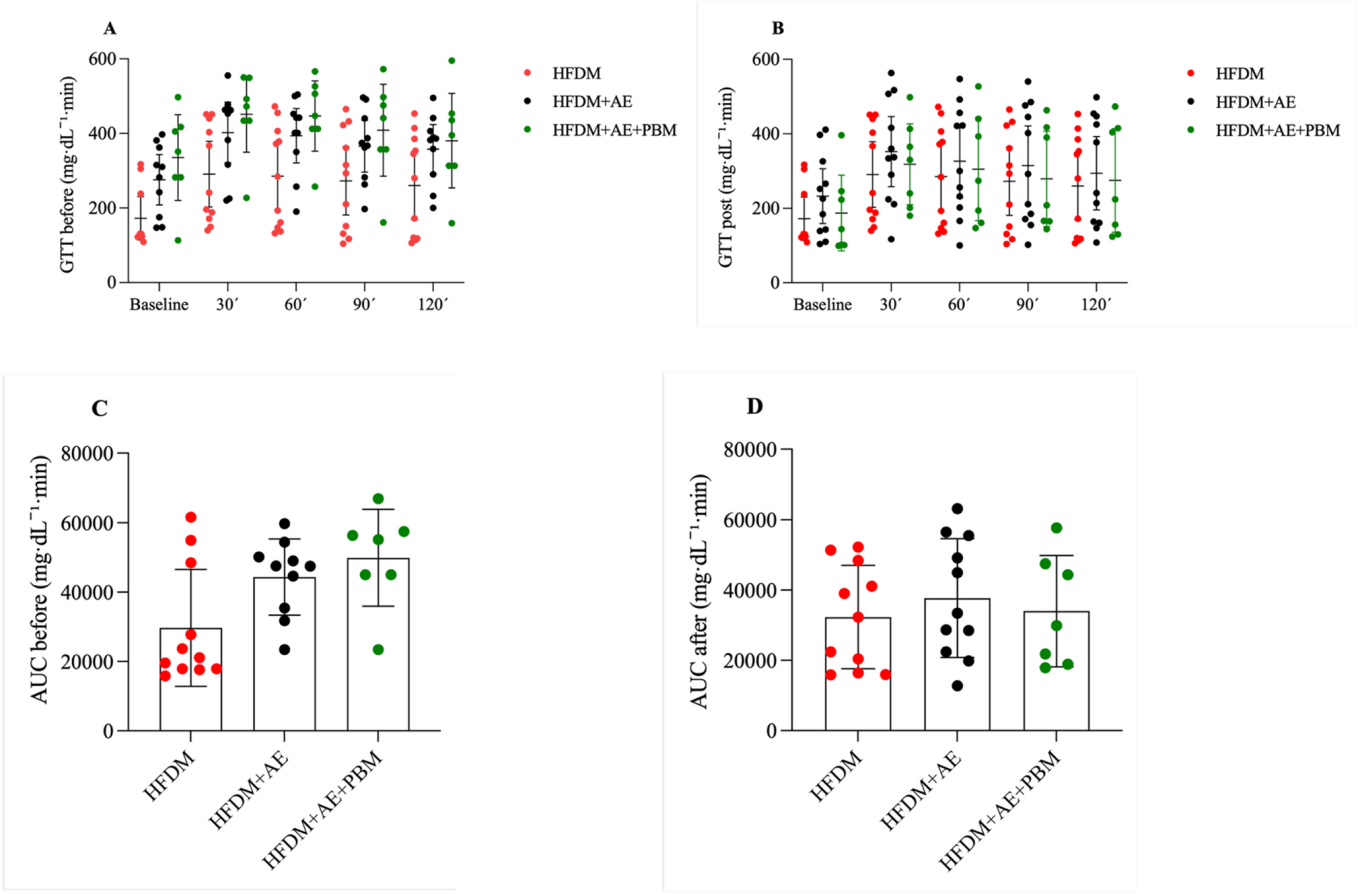
Glucose tolerance test (GTT) in rats with type 2 diabetes mellitus and heart failure. Panels **A** and **B** show blood glucose concentrations (mg·dL⁻¹) during the GTT at baseline and after the intervention, respectively. Panels **C** and **D** present the area under the curve (AUC; mg·dL⁻¹·min), calculated from glucose values obtained at 0, 30, 60, 90, and 120 min before and after the intervention, respectively. Individual data points represent each animal, and bars indicate mean values with 95% confidence intervals. Statistical significance was set at *p* < 0.05. Group sizes were HFDM (*n* = 11), heart failure associated with type 2 diabetes mellitus; HFDM + AE (*n* = 12), heart failure associated with type 2 diabetes mellitus treated with aerobic exercise; and HFDM + AE+PBM (*n* = 7), heart failure associated with type 2 diabetes mellitus treated with aerobic exercise combined with photobiomodulation

**Fig. 4:**
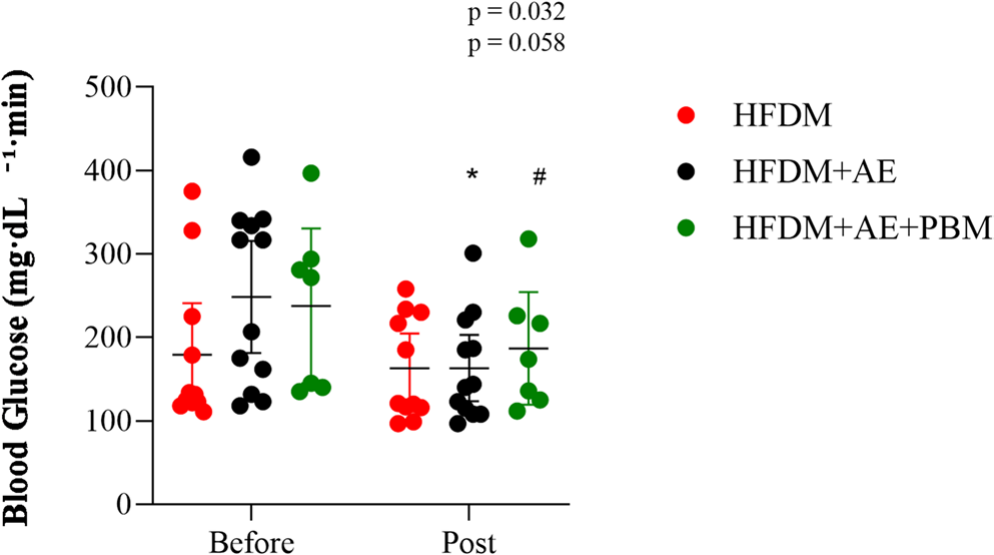
Fasting blood glucose levels before and after the intervention in rats with type 2 diabetes mellitus and heart failure. Blood glucose concentrations (mg/dL) were assessed before and after the intervention period in the HFDM, HFDM + AE, and HFDM + AE+PBM groups. Individual data points represent each animal, and symbols with error bars indicate mean ± standard deviation. Statistical analysis was performed using two-way repeated-measures ANOVA followed by Tukey’s post hoc test. Statistical significance was set at *p* < 0.05. Group sizes were HFDM (*n* = 11), heart failure associated with type 2 diabetes mellitus; HFDM + AE (*n* = 12), heart failure associated with type 2 diabetes mellitus treated with aerobic exercise; and HFDM + AE+PBM (*n* = 7), heart failure associated with type 2 diabetes mellitus treated with aerobic exercise combined with photobiomodulation. *p = 0.032 vs. pre-intervention within the HFDM + AE group; #*p* = 0.058 vs. pre-intervention within the HFDM + AE+PBM group

Glucose tolerance test curves (Fig. 3) did not differ significantly among groups at either baseline or after the intervention. Consistent with this finding, analysis of the area under the curve (AUC) with 95% confidence intervals revealed no between-group differences (*p* > 0.05). In contrast, fasting glucose levels decreased significantly after the intervention in the HFDM + AE group (*p* = 0.032) and showed a strong trend toward in the HFDM + AE+PBM group (*p* = 0.058), while remaining unchanged in the HFDM group (Fig. 4).

### Echocardiographic parameters

Table 2 summarizes the echocardiographic parameters. Left ventricular EF did not differ among groups (HFDM: 38.3 ± 7.8%; HFDM + AE: 44.2 ± 6.3%; HFDM + AE+PBM: 36.8 ± 8.2%; *p* = 0.062), although a trend toward higher EF was observed in the HFDM + AE group. Fractional shortening also did not differ among groups (*p* = 0.085). With respect to ventricular structural remodeling, LV posterior wall thickness in diastole (LVPWd) and systole (LVPWs) were significantly lower in the HFDM + AE and HFDM + AE+PBM groups compared with the HFDM (no intervention) group (*p* = 0.0001).

**Table 2:** Echocardiographic variables measured after completion of the interventions

Measurements	HFDM (*n* = 11)	HFDM + AE (*n* = 12)	HFDM + AE+PBM (*n* = 7)	*P* –value
EF (%)	38.3 ± 7.8	44.2 ± 6.3	36.8 ± 8.2	0.062
IVSd (mm)	0.16 ± 0.05	0.14 ± 0.02	0.16 ± 0.03	0.614
LVESd (mm)	0.86 ± 0.1	0.85 ± 0.1	0.77 ± 0.1	0.185
LVPWs (mm)	0.27 ± 0.1	0.14 ± 0.03*	0.16 ± 0.03#	0.0001
IVSs (mm)	0.19 ± 0.07	0.21 ± 0.03	0.19 ± 0.02	0.510
LVEDd (mm)	0.91 ± 0.09	0.93 ± 0.45	0.94 ± 0.10	0.730
LVPWd (mm)	0.24 ± 0.06	0.15 ± 0.03*	0.15 ± 0.02#	0.0001
FS (%)	15.8 ± 3.6	17.1 ± 3.5	13.5 ± 2.1	0.085
RWT	0.32 ± 0.03	0.29 ± 0.03	0.32 ± 0.05	0.089
E’ (cm s^− 1^)	0.43 ± 0.10	0.48 ± 0.10	0.43 ± 0.08	0.372
A’ (cm s^− 1^)	0.24 ± 0.03	0.25 ± 0.02	0.22 ± 0.05	0.257
E/A	1.78 ± 0.38	2.34 ± 0.71	2.01 ± 0.51	0.07

Values are presented as mean ± standard deviation. Statistical comparisons were performed using one-way ANOVA followed by the Bonferroni post hoc test. Group sizes were as follows: HFDM (*n* = 11), heart failure associated with type 2 diabetes mellitus; HFDM + AE (*n* = 12), heart failure associated with type 2 diabetes mellitus treated with aerobic exercise; and HFDM + AE+PBM (*n* = 7), heart failure associated with type 2 diabetes mellitus treated with aerobic exercise combined with photobiomodulation. Abbreviations: EF, ejection fraction (%); HR, heart rate (beats/min); IVSd, interventricular septal thickness in diastole (mm); IVSs, interventricular septal thickness in systole (mm); LVEDd, left ventricular end-diastolic diameter (mm); LVESd, left ventricular end-systolic diameter (mm); LVPWd, left ventricular posterior wall thickness in diastole (mm); LVPWs, left ventricular posterior wall thickness in systole (mm); FS, fractional shortening (%); RWT, relative wall thickness; E′, early diastolic peak velocity (cm·s⁻¹); A′, late diastolic peak velocity (cm·s⁻¹); E/A, ratio of early to late mitral inflow velocities. *p < 0.0001 vs. HFDM (HFDM + AE); #*p* = 0.009 vs. HFDM (HFDM + AE+PBM)

### Haemodynamic

Table 3 summarizes haemodynamic parameters. The + dP/dtmax was significantly higher in HFDM + AE group compared with the both the HFDM group (*p* < 0.001) and the HFDM + AE+PBM group (*p* = 0.009). Similarly, the -dP/dtmin was significantly improved in the HFDM + AE group relative to the HFDM (no intervention) group (*p* < 0.001).

**Table 3:** Invasive hemodynamic variables

Measurements	HFDM (*n* = 11)	HFDM + AE (*n* = 12)	HFDM + AE+PBM (*n* = 7)	*P*-value
LVEDP (mmHg)	12.3 ± 12.1	12.6 ± 3.3	8.9 ± 4.6	0.102
LVSP (mmHg)	90.6 ± 21.4	103.2 ± 22.7	99.5 ± 10.2	0.142
+dP/dt_max_ (mmHg/s)	2109.6 ± 19.2	3599.1 ± 805.5*#	2792.2 ± 895	0.001
-dP/dt_min_ (mmHg/s)	−1742.3 ± 1025.5	−2604.5 ± 747.3*	−2287.2 ± 1161	0.001

Values are presented as mean ± standard deviation. Statistical comparisons were performed using one-way ANOVA followed by the Bonferroni post hoc test. Group sizes were as follows: HFDM (*n* = 11), heart failure associated with type 2 diabetes mellitus; HFDM + AE (*n* = 12), heart failure associated with type 2 diabetes mellitus treated with aerobic exercise; and HFDM + AE+PBM (*n* = 7), heart failure associated with type 2 diabetes mellitus treated with aerobic exercise combined with photobiomodulation. Abbreviations: EF, ejection fraction (%); HR, heart rate (beats/min); IVSd, interventricular septal thickness in diastole (mm); IVSs, interventricular septal thickness in systole (mm); LVEDd, left ventricular end-diastolic diameter (mm); LVESd, left ventricular end-systolic diameter (mm); LVPWd, left ventricular posterior wall thickness in diastole (mm); LVPWs, left ventricular posterior wall thickness in systole (mm); FS, fractional shortening (%); RWT, relative wall thickness; E′, early diastolic peak velocity (cm·s⁻¹); A′, late diastolic peak velocity (cm·s⁻¹); E/A, ratio of early to late mitral inflow velocities. *p < 0.0001 vs. HFDM (HFDM + AE); #*p* = 0.009 vs. HFDM (HFDM + AE+PBM)

### Functional capacity

**Fig. 5:**
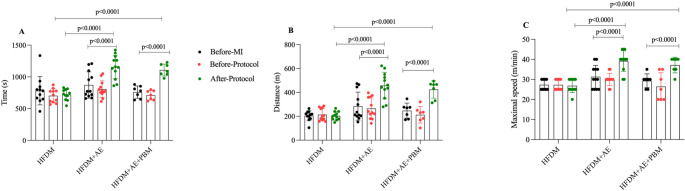
Functional capacity assessed by treadmill exercise testing in rats with type 2 diabetes mellitus and heart failure. Panels **A**–**C** show exercise performance measured as time to exhaustion (s), running distance (m), and maximal running speed (m/min), respectively, assessed at the pre–myocardial infarction, pre-intervention, and post-intervention time points. Individual data points represent each animal, and bars indicate mean values ± standard deviation. Statistical analysis was performed using a generalized linear model (GLM) with Bonferroni correction for multiple comparisons. Statistical significance was set at *p* < 0.05. Group sizes were HFDM (*n* = 11), heart failure associated with type 2 diabetes mellitus; HFDM + AE (*n* = 12), heart failure associated with type 2 diabetes mellitus treated with aerobic exercise; and HFDM + AE+PBM (*n* = 7), heart failure associated with type 2 diabetes mellitus treated with aerobic exercise combined with photobiomodulation. Horizontal bars indicate statistically significant differences between time points (pre- vs. post-intervention) and between groups in the post-intervention evaluation, as indicated by the p-values displayed in the graphs (*p* < 0.0001 in panels **A**–**C**)

Baseline functional test performance was comparable among groups. After intervention, both the HFDM + AE and HFDM + AE+PBM groups exhibited significantly longer exercise duration (Fig. 5A), greater running distance (Fig. 5B), and higher maximal speed (Fig. 5C) compared to baseline (*p* < 0.0001) and relative to the HFDM (no intervention) group.

### Inflammatory cytokines

Plasma, LV, and gastrocnemius levels of TNF-α, IL-1β, and IL-10 did not differ significantly among groups (*p* > 0.05). These data are summarized in Table 4, which presents TNF-α, IL-10, and IL-1β concentrations in the left ventricle, plasma, and gastrocnemius muscle.

**Table 4:** Concentrations of TNF-α, IL-10, and IL-1β in the left ventricle, plasma, and gastrocnemius muscle

Measurements	HFDM (*n* = 11)	HFDM + AE (*n* = 12)	HFDM + AE+PBM (*n* = 7)	*P*-value
TNF-α in LV (pg/mg of protein)	68.5 ± 44.6	53.4 ± 24.2	51.9 ± 24.9	0.534
TNF-α in plasma (pg/mL)	19.9 ± 5.2	17.5 ± 2.2	21 ± 5.4	0.371
TNF-α in gastrocnemius muscle (pg/mg of protein)	52.6 ± 25.3	59.8 ± 23.7	46.8 ± 9.6	0.490
IL-10 in LV (pg/mg of protein)	32 ± 18	39.3 ± 23.7	28.2 ± 15.7	0.528
IL-10 in plasma (pg/mL)	13.9 ± 9	8.9 ± 2.5	6.74 ± 1.9	0.061
IL-10 in gastrocnemius muscle (pg/mg of protein)	20.7 ± 9.4	28.9 ± 15.7	23 ± 7.6	0.334
IL-1β in LV (pg/mL)	3317 ± 501.3	3677 ± 338.2	3604 ± 749.5	0.417
IL-1β in gastrocnemius muscle (pg/mg of protein)	1157 ± 572.3	1521 ± 764.2	1677 ± 1121	0.504

Values are presented as mean ± standard deviation. Statistical comparisons were performed using one-way ANOVA followed by Tukey’s post hoc test. Group sizes were as follows: HFDM (*n* = 11), heart failure associated with type 2 diabetes mellitus; HFDM + AE (*n* = 12), heart failure associated with type 2 diabetes mellitus treated with aerobic exercise; and HFDM + AE+PBM (*n* = 7), heart failure associated with type 2 diabetes mellitus treated with aerobic exercise combined with photobiomodulation. Abbreviations: LV, left ventricle; TNF-α, tumour necrosis factor-α; IL-10, interleukin-10; IL-1β, interleukin-1β. Cytokine concentrations are expressed as pg/mg of protein for tissue samples and pg/mL for plasma

### Oxidative stress and antioxidant enzymes

**Fig. 6:**
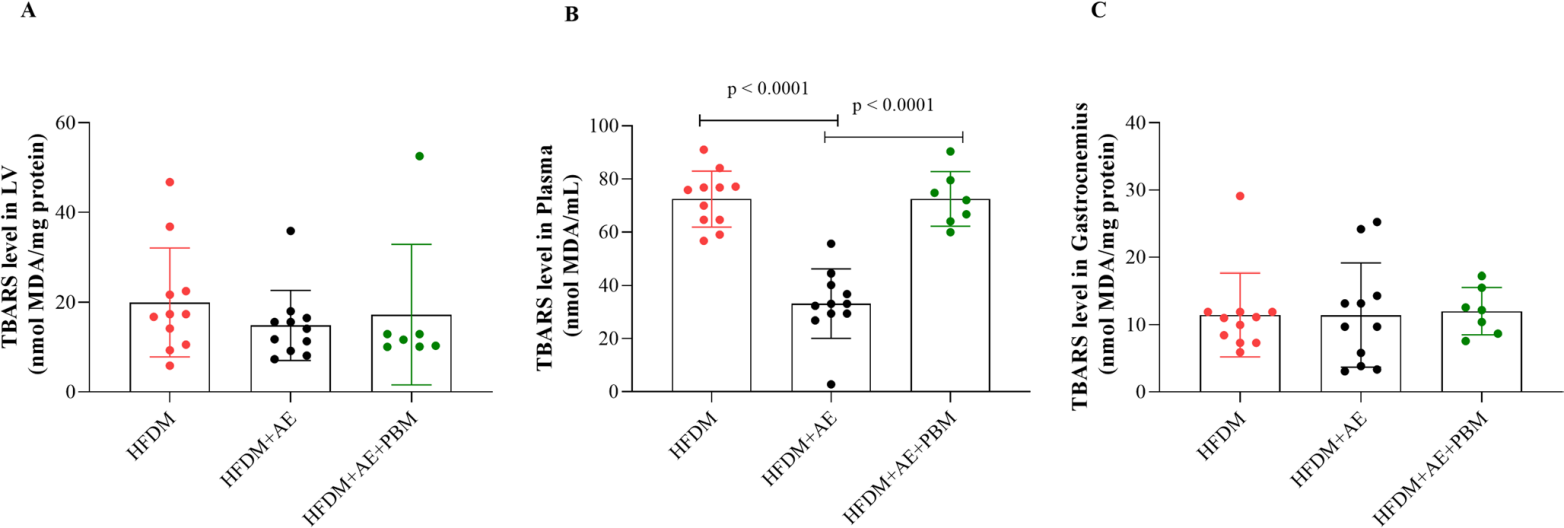
Lipid peroxidation assessed by thiobarbituric acid–reactive substances (TBARS) in rats with type 2 diabetes mellitus and heart failure. Panels A–C show TBARS concentrations measured in the left ventricle (nmol malondialdehyde [MDA]/mg protein), plasma (nmol MDA/mL), and gastrocnemius muscle (nmol MDA/mg protein), respectively. Individual data points represent each animal, and bars indicate mean values ± standard deviation. Statistical analysis was performed using one-way ANOVA followed by Tukey’s post hoc test. Statistical significance was set at *p* < 0.05. Group sizes were HFDM (*n* = 11), heart failure associated with type 2 diabetes mellitus; HFDM + AE (*n* = 12), heart failure associated with type 2 diabetes mellitus treated with aerobic exercise; and HFDM + AE+PBM (*n* = 7), heart failure associated with type 2 diabetes mellitus treated with aerobic exercise combined with photobiomodulation. Horizontal bars indicate statistically significant between-group differences, as denoted by the p-values shown in the graphs (*p* < 0.0001 in panel B for HFDM + AE vs. HFDM and HFDM + AE+PBM)

TBARS: Plasma TBARS levels were significantly lower in the HFDM + AE group compared with both the HFDM and HFDM + AE+PBM groups (*p* < 0.0001; Fig. 6B). In contrast, TBARS levels in LV and gastrocnemius muscle did not differ among groups (Fig. 6A and C).

**Fig. 7:**
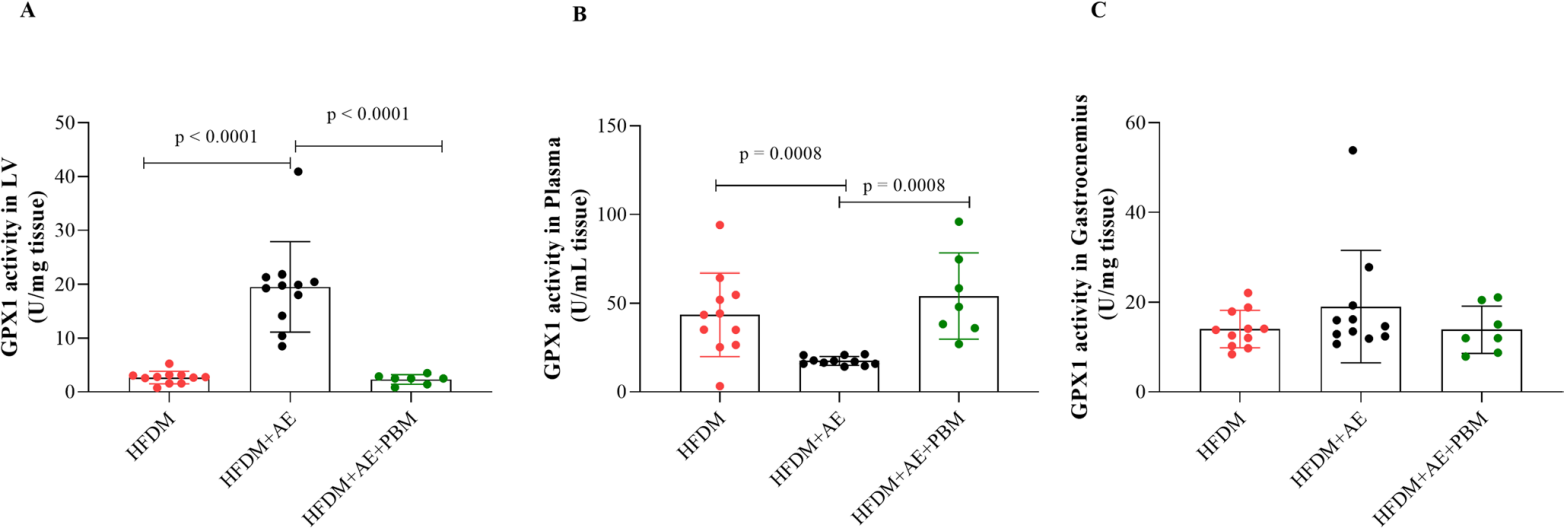
Glutathione peroxidase 1 (GPX1) activity in rats with type 2 diabetes mellitus and heart failure. Panels A–C show GPX1 activity measured in the left ventricle, plasma, and gastrocnemius muscle, expressed as units per milligram of tissue (U/mg tissue) for tissue samples and units per millilitre (U/mL) for plasma. Individual data points represent each animal, and bars indicate mean values ± standard deviation. Statistical analysis was performed using one-way ANOVA followed by Tukey’s post hoc test. Statistical significance was set at *p* < 0.05. Group sizes were HFDM (*n* = 11), heart failure associated with type 2 diabetes mellitus; HFDM + AE (*n* = 12), heart failure associated with type 2 diabetes mellitus treated with aerobic exercise; and HFDM + AE+PBM (*n* = 7), heart failure associated with type 2 diabetes mellitus treated with aerobic exercise combined with photobiomodulation. Horizontal bars indicate statistically significant between-group differences, as denoted by the p-values shown in the graphs (*p* < 0.0001 in panel A and *p* = 0.0008 in panel B for HFDM + AE vs. HFDM and HFDM + AE+PBM)

GPX1 activity: GPX1 activity was significantly increased in LV of the HFDM + AE group compared with both other groups (*p* < 0.0001; Fig. 7A). In plasma, GPX1 activity was significantly lower in the HFDM + AE group than in the HFDM and HFDM + AE+PBM groups (*p* = 0.0008; Fig. 7B). No differences were observed in gastrocnemius muscle GPX1 activity among groups (Fig. 7C).

**Fig. 8:**
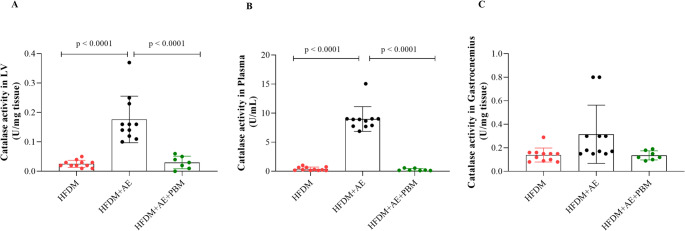
Catalase (CAT) activity in rats with type 2 diabetes mellitus and heart failure. Panels A–C show CAT activity measured in the left ventricle, plasma, and gastrocnemius muscle, expressed as units per milligram of tissue (U/mg tissue) for tissue samples and units per millilitre (U/mL) for plasma. Individual data points represent each animal, and bars indicate mean values ± standard deviation. Statistical analysis was performed using one-way ANOVA followed by Tukey’s post hoc test. Statistical significance was set at *p* < 0.05. Group sizes were HFDM (*n* = 11), heart failure associated with type 2 diabetes mellitus; HFDM + AE (*n* = 12), heart failure associated with type 2 diabetes mellitus treated with aerobic exercise; and HFDM + AE+PBM (*n* = 7), heart failure associated with type 2 diabetes mellitus treated with aerobic exercise combined with photobiomodulation. Horizontal bars indicate statistically significant between-group differences, as denoted by the p-values shown in the graphs (*p* < 0.0001 in panels A and B for HFDM + AE vs. HFDM and HFDM + AE+PBM)

CAT activity: CAT activity was significantly elevated in both the LV and plasma of the HFDM + AE group compared with the HFDM and HFDM + AE+PBM groups (*p* < 0.0001; Fig. 8A and B). No significant group differences were detected in gastrocnemius muscle CAT activity (Fig. 8C).

## Discussion

This study investigated the combined effects of AE and PBM in a rat model of HF associated with DM, a combination that has not been previously explored. We demonstrated that AE significantly improved cardiac function by reducing left ventricular wall thickness, enhancing contractility (+ dP/dtmax and -dP/dtmin), and increasing exercise tolerance. In this model, AE exerted its most pronounced effects on functional performance and left ventricular structural remodeling, hemodynamic indices and markers related to redox balance, particularly in plasma and LV tissue. These findings indicate that cardiorespiratory performance and redox regulation are highly responsive to aerobic training in the context of HFDM. In addition, AE improved fasting glycaemia and was associated with a trend toward reduced systemic oxidative stress, as evidenced by lower TBARS levels and increased GPX1 and CAT activities. In contrast, PBM applied after AE, did not confer additional benefits beyond those achieved with AE alone.

The HFDM model was confirmed by an infarct size > 30%, LVEF < 50%, and persistent hyperglycaemia, in agreement with previous studies [5, 33]. The beneficial effects of AE on ventricular remodelling and haemodynamic function are consistent with prior evidence demonstrating that exercise training attenuates adverse cardiac adaptations and improves functional performance in HF models [23, 34].

Regarding metabolic outcomes, AE improved fasting glycaemia but did not significantly alter glucose tolerance (GTT). This divergence likely reflects the involvement of distinct physiological domains: fasting glycaemia is primarily determined by hepatic glucose output, whereas glucose tolerance depends largely on peripheral insulin sensitivity. Exercise-induced reductions in fasting glycaemia may be mediated by decreased hepatic gluconeogenesis through pathways involving PI3K/Akt signaling, as suggested by previous studies [35, 36]. In contrast, persistent peripheral insulin resistance in the HFDM model may explain the unchanged GTT results [37]. The absence of direct assessments of insulin sensitivity, such as the insulin tolerance test (ITT) or the homeostatic model assessment of insulin resistance (HOMA-IR), limits mechanistic interpretation. Nevertheless, our findings are consistent with prior reports indicating that AE more reliably improves basal glycaemia control than dynamic glucose disposal in HFDM contexts [37]. Similarly, AE did not significantly modify circulating inflammatory cytokine concentrations (TNF-α, IL-1β, IL-10), although directional changes were observed. This selective responsiveness may reflect persistent metabolic inflexibility and low-grade systemic inflammatory signaling characteristic of HFDM, which may require longer intervention periods or combined metabolic therapies to achieve meaningful modulation.

Antioxidant adaptations were evident, as indicated by increased GPX1 and CAT activities and reduced TBARS levels. These redox adaptations occurred in parallel with improvements in cardiac function and are consistent with the established role of AE in modulating oxidative balance; however, no direct causal relationship can be inferred from the present data. These findings are consistent with previous evidence suggesting that AE activates antioxidant responses through pathways associated with Nrf2 signaling, although this pathway was not directly assessed in the current study [38, 39]. The apparent redistribution of GPX1 and CAT activity toward cardiac tissue suggests a preferential antioxidant adaptation in the myocardium in the context of HFDM-related oxidative injury [40]. Elevated TBARS are widely recognized as markers of lipid peroxidation [41], and their directional reduction following AE supports attenuation of lipid peroxidation–associated oxidative stress, which has been linked to improved mitochondrial efficiency in previous studies [42, 43].

In contrast, PBM failed to augment the benefits of AE. Several explanations may account for this finding: (1) a ceiling effect, whereby AE alone maximised physiological adaptations; (2) mitochondrial dysfunction intrinsic to DM, potentially limiting PBM responsiveness; and (3) high sensitivity of PBM to parameters such as wavelength, dose, application site, and timing [31, 44]. Although PBM has been reported to improve performance and redox balance in isolated HF or DM models [16, 17], the present findings suggest that, in the combined HFDM condition, its effects are context-dependent and may be attenuated.

The lack of additive benefit when PBM was combined with AE may also reflect interference with exercise-induced redox signaling, which involves transient reactive oxygen species elevations that are critical for adaptive antioxidant responses. Additionally, the gastrocnemius muscle was selected as a peripheral target to explore potential systemic effects; however, localized irradiation of skeletal muscle may not replicate the benefits observed with direct cardiac PBM application [45].

Interestingly, in our data (Figs. 6, 7 and 8), suggest that the addition of PBM may have attenuated or abolished some of the benefits induced by aerobic exercise. This observation should be interpreted with caution, as it may be related to the biphasic dose–response characteristic of PBM, whereby specific parameter combinations can interfere with exercise-induced signaling, particularly redox-mediated adaptations. Because aerobic exercise relies on transient increases in reactive oxygen species to activate endogenous antioxidant defenses, immediate post-exercise PBM application may have blunted this adaptive signaling cascade.

Moreover, the complex pathophysiological context of HFDM, characterized by mitochondrial dysfunction and altered inflammatory signaling, may further amplify this antagonistic interaction. The smaller sample size in the AE + PBM group (*n* = 7) should also be considered as a potential contributor to this finding. Finally, circulating inflammatory cytokines (TNF-α, IL-1β, and IL-10) were not significantly modulated by either AE or PBM at the single time point assessed. This pattern may reflect the dynamic and transient nature of inflammatory cytokine responses, which are often missed in the absence of serial measurements [46]. In contrast, antioxidant adaptations tend to be more sustained, which may explain why redox-related responses were more readily detected in the present study.

Taken together, these findings help delineate the physiological domains most responsive to aerobic exercise in the HFDM context and indicate that PBM, under the parameters employed, does not potentiate these adaptations. The absence of PBM-only and sham PBM control groups limits the ability to isolate the independent effects of PBM. Future studies should therefore explore alternative PBM dosing strategies, timing relative to exercise, and tissue-specific targeting to determine whether synergistic effects with aerobic exercise can be achieved.

Overall, our findings confirm the cardiometabolic and antioxidant benefits of AE in HFDM, which occurred in parallel and should be interpreted as associated rather than causally linked outcomes. At the same time, the results highlight the limited additional impact of PBM under the conditions tested. Future studies should refine PBM protocols, including dose, application site, and timing, incorporate direct assessments of insulin sensitivity, and employ multiple sampling time points to better elucidate the interactions among AE, PBM, oxidative stress, and inflammation in HFDM.

## Limitations

Several limitations should be acknowledged. Oxidative stress assessment relied primarily on TBARS, which reflects lipid peroxidation but lacks specificity for other oxidative damage pathways and therefore does not capture global oxidative injury. Circulating inflammatory cytokines (TNF-α, IL-6, and IL-10) were measured at a single time point, potentially missing transient responses induced by AE or PBM. Insulin sensitivity was not directly assessed (e.g., insulin levels, HbA1c, insulin tolerance test, or HOMA-IR), limiting mechanistic interpretation of the dissociation between fasting glycaemia and glucose tolerance.

Echocardiographic and haemodynamic measurements were performed under anaesthesia, which may influence cardiac loading conditions; however, all groups were evaluated under identical protocols. In addition, sample size was reduced due to attrition, particularly in the HFDM + AE+PBM group, which may have limited statistical power for some outcomes. This study also did not include a non-HFDM control group, as the primary aim was to compare therapeutic interventions within the HFDM phenotype. Although this limits comparisons with normal physiological values, it preserves internal validity and aligns with ethical principles to minimize unnecessary animal use.

Finally, the absence of PBM-only and sham PBM groups precludes isolation of the independent effects of PBM. Accordingly, the present findings should be interpreted as reflecting the additive effects of PBM when combined with aerobic exercise under the conditions tested.

## Conclusions

Aerobic exercise improved cardiac function, glycaemic control, and markers of lipid peroxidation and antioxidant defense in rats with heart failure associated with diabetes mellitus. In contrast, PBM did not provide additional benefits under the conditions applied, potentially due to disease-specific mitochondrial dysfunction or limitations related to dosing parameters. Further studies are warranted to optimize PBM protocols and to better define its therapeutic role in HFDM. 

## Data Availability

All data supporting the findings of this study are included in the article or are available from the corresponding author upon reasonable request.

## Abbreviations

AE: Aerobic Exercise
Akt: Protein Kinase B
ARRIVE: Animal Research: Reporting of In Vivo Experiments
ATP: Adenosine Triphosphate
AUC: Area Under the Curve
CAT: Catalase
DM: Diabetes Mellitus
dP/dtmax: Maximum Rate of Left Ventricular Pressure Rise
dP/dtmin: Minimum Rate of Left Ventricular Pressure Decline
EF: Ejection Fraction
FS: Fractional Shortening
GaAlAs: Gallium–Aluminum–Arsenide
GPX1: Glutathione Peroxidase 1
GTT: Glucose Tolerance Test
HF: Heart Failure
HFDM: Heart Failure Associated with Diabetes Mellitus
HOMA: IR–Homeostatic Model Assessment of Insulin Resistance
H₂O₂: Hydrogen Peroxide
IL: 1β–Interleukin–1 Beta
IL: 6–Interleukin–6
IL: 10–Interleukin–10
i.p.: Intraperitoneal
ITT: Insulin Tolerance Test
LV: Left Ventricle
LVEDP: Left Ventricular End–Diastolic Pressure
LVPWd: Left Ventricular Posterior Wall in Diastole
LVPWs: Left Ventricular Posterior Wall in Systole
MAP: Multiplex Assay Panel
MI: Myocardial Infarction
NADPH: Nicotinamide Adenine Dinucleotide Phosphate (Reduced Form)
Nrf2: Nuclear Factor Erythroid 2–Related Factor 2
PBM: Photobiomodulation
PI3K: Phosphoinositide 3–Kinase
ROS: Reactive Oxygen Species
RWT: Relative Wall Thickness
RV: Right Ventricle
SOD: Superoxide Dismutase
SPSS: Statistical Package for the Social Sciences
STZ: Streptozotocin
TBARS: Thiobarbituric Acid Reactive Substances
TNF– α: Tumor Necrosis Factor Alpha
